# Reinvestigating Tumor–Ventricle Relationship of Craniopharyngiomas With Predominantly Ventricular Involvement: An Endoscopic Endonasal Series Based on Histopathological Assessment

**DOI:** 10.3389/fonc.2021.740410

**Published:** 2021-12-03

**Authors:** Jun Fan, Yi Liu, Chaohu Wang, Zhanpeng Feng, Jun Pan, Yuping Peng, Junxiang Peng, Yun Bao, Jing Nie, Binghui Qiu, Songtao Qi

**Affiliations:** Department of Neurosurgery, Nanfang Hospital, Southern Medical University, Guangzhou, China

**Keywords:** craniopharyngioma, endonasal, endoscopic, histology, intraventricular, pituitary surgery, third ventricle

## Abstract

**Objective:**

Craniopharyngiomas (CPs) predominantly involving the third ventricle were commonly termed “intraventricular” lesions. The aim of this study was to clarify the anatomical relationship between the tumor and the third ventricle by both surgical and histological investigation.

**Methods:**

A retrospective review of primarily resected CPs by endoscopic endonasal surgery was performed. CPs with predominantly ventricular involvement were selected for study inclusion by preoperative imaging. The surgical procedure of each case was reviewed. The wholly removed tumor specimens were histologically analyzed, in all cases, to investigate the tumor–third ventricle relationship using hematoxylin and eosin, immunochemical, and immunofluorescence staining.

**Results:**

Twenty-six primary CPs predominantly involving the third ventricle were selected from our series of 223 CPs treated by endoscopic endonasal surgery between January 2017 and March 2021. Gross-total resection was achieved in 24 (92.3%) of 26 patients, with achievement of near-total resection in the remaining patients. A circumferential layer of stretched third ventricle floor was identified surrounding the tumor capsule, which could be peeled off easily from the ventricle floor remnants at most areas of the plane of tumor attachment. Some portions of the tumor capsule tightly adhered to the third ventricle floor were removed together with the floor. A breach of various size was observed at the third ventricle floor after tumor removal in most cases, the floor remaining intact in only two cases (7.7%). Histological examination on marked portions of tumor capsule showed that the pia mater was frequently detected at most of the tumor–brain interface, except at the antero-frontal border of tumor contacting with the third ventricle floor. At this point, a layer of gliosis with various thickness was observed between the tumor and the neural tissue of the third ventricle floor.

**Conclusion:**

CPs with predominantly ventricular involvement should be considered as lesions with an extraventricular, epi-pia topography rather than “intraventricular” or “subpial” topography. Accurate understanding of the relationship between the third ventricle and such tumors would predict the circumferential cleavage plane of dissection, and remind neurosurgeons of performing dissection along the safe surgical plane to achieve total tumoral resection with minimizing hypothalamic damage.

## Introduction

Craniopharyngiomas (CPs) are benign tumors believed to originate from ectodermal remnants of the craniopharygeal duct, accounting for 2% to 5% of all intracranial tumors (1). These tumors may arise at any point along the hypothalamus–pituitary axis, intrasellar or suprasellar (2, 3). CPs predominantly involving the third ventricle remain among the most challenging to form a surgical perspective due to their upward extension against the third ventricle and their close relationship with the hypothalamus. An accurate understanding of the topographical relationships between the CPs and the third ventricle floor/hypothalamus is essential for proper, safe surgical planning or manipulation.

CPs predominantly involving the third ventricular compartment were commonly described as “intraventricular” or “third ventricle” lesions in most publications, and these tumors were thereby classified into “intraventricular” subset by many authors in their classification system (4–14). Since the term “intraventricular” specifically denote lesions that arise from the brain parenchyma and grow exophytically into the ventricular system, “intraventricular” CPs were thought to originally develop within the neural tissue of the third ventricle floor and progressively grow into the third ventricle leaving an intact third ventricle floor below (15, 16). However, the theory is in conflict with the universally accepted hypothesis that CPs arise from ectodermal remnants of the craniopharygeal duct. On the other hand, previous classification systems including the “intraventricular” subset of CPs were established based on tumor–third ventricle relationships observed on autopsies, surgical procedures, and/or neuroradiological studies (7, 11, 17, 18). Accurate histological evidence of the CP boundaries contacting the third ventricle floor and the third ventricle walls is lacking in most reports on intraventricular CPs to unequivocally define the strict “intraventricular” category.

Endoscopic endonasal surgery use a corridor along the hypothalamic–pituitary axis and allows direct visualization of retrochiasmatic compartment, especially for the ventral area of the third ventricle (17, 19, 20). Accordingly, this approach provides more precise information for illuminating the tumor–third ventricle relationships than traditional microscopic transcranial approach. In this series, we retrospectively reviewed surgical procedures of CPs with predominantly ventricular involvement by endoscopic endonasal approach, and made comprehensive histological evaluation of tumor–third ventricle interface on these tumors. The aim of this study is to reveal the real relationships between the tumor and the third ventricle with more convincing evidence, and to provide useful information for neurosurgeons when managing such tumors with appropriate surgical planning and minimizing the risk of damage to the hypothalamus.

## Materials and Methods

### Patient Selection and Perioperative Assessment

**Figure 1:**
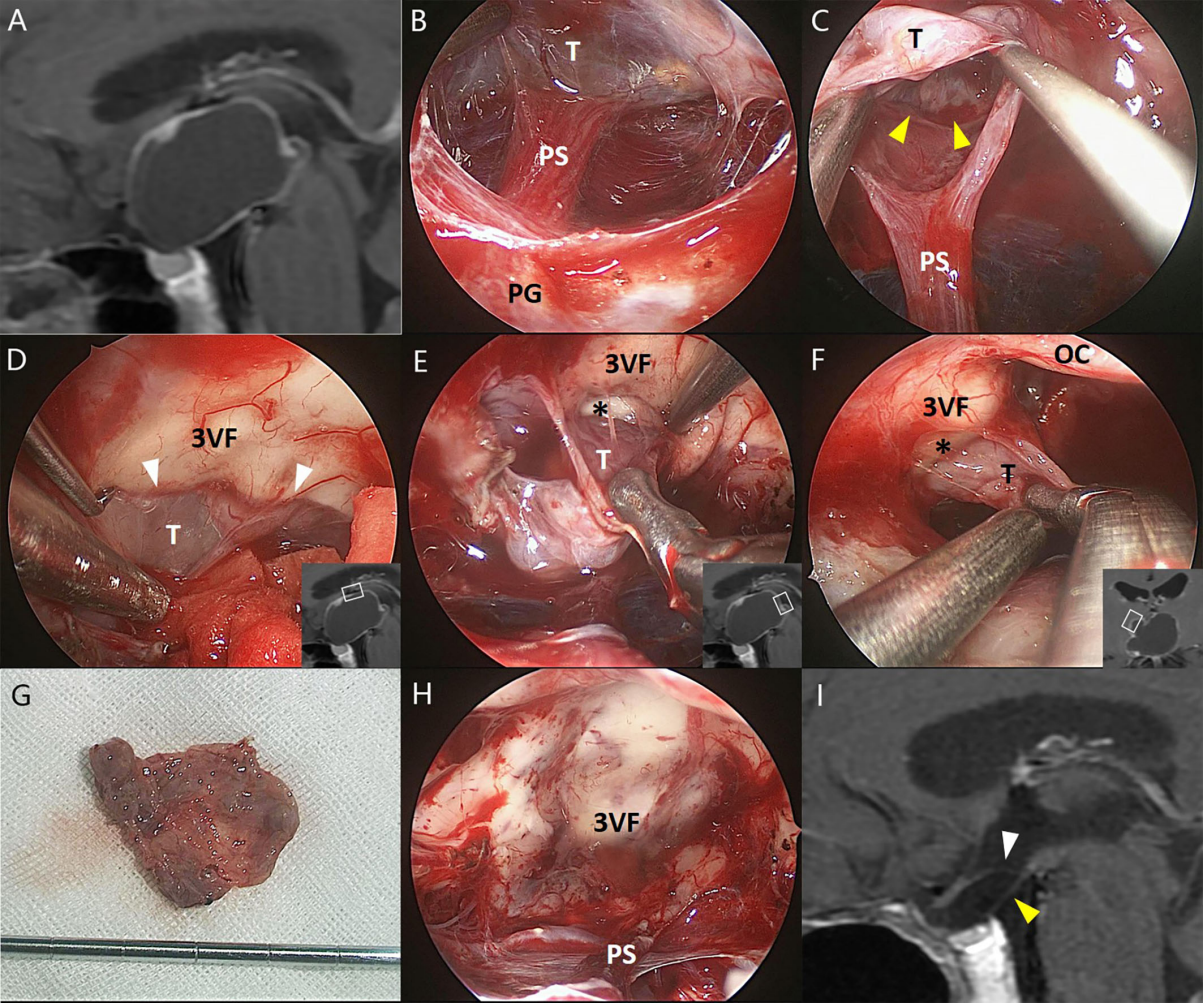
Illustrative endonasal case 1 clarifying the tumor–ventricle relationship. In **(A)**, preoperative MRI shows that the CP mainly occupies the third ventricle chamber. In **(B)**, intraoperative photograph indicates the intact pituitary stalk beneath the tumor. In **(C)**, a clear plane of dissection (yellow arrowhead) was identified at the tumor–infundibulum interface. In **(D)**, the top portion of the tumor was easily stripped away from the third ventricle floor through a cleavage pial plane (white arrowhead). In **(E, F)**, the pia mater was absent at some areas of the tumor–ventricle interface (asterisk) while a safe plane of surgical dissection could still be identified to perform the total tumoral resection without trespassing the third ventricle floor. In **(G)**, the tumor was removed en bloc after circumferential dissection. In **(H)**, the third ventricle floor as well as the pituitary stalk remained intact without any defect after total tumoral removal, suggesting the extraventricular rather than the “intraventricular” topography of such tumors. In **(I)**, postoperative MRI confirms the total removal of the tumor and the integrity of the third ventricle floor (white arrowhead) and the pituitary gland (yellow arrowhead). 3VF, the third ventricle floor; OC, optic chiasm; PG, pituitary gland; PS, pituitary stalk; T, tumor.

**Figure 2:**
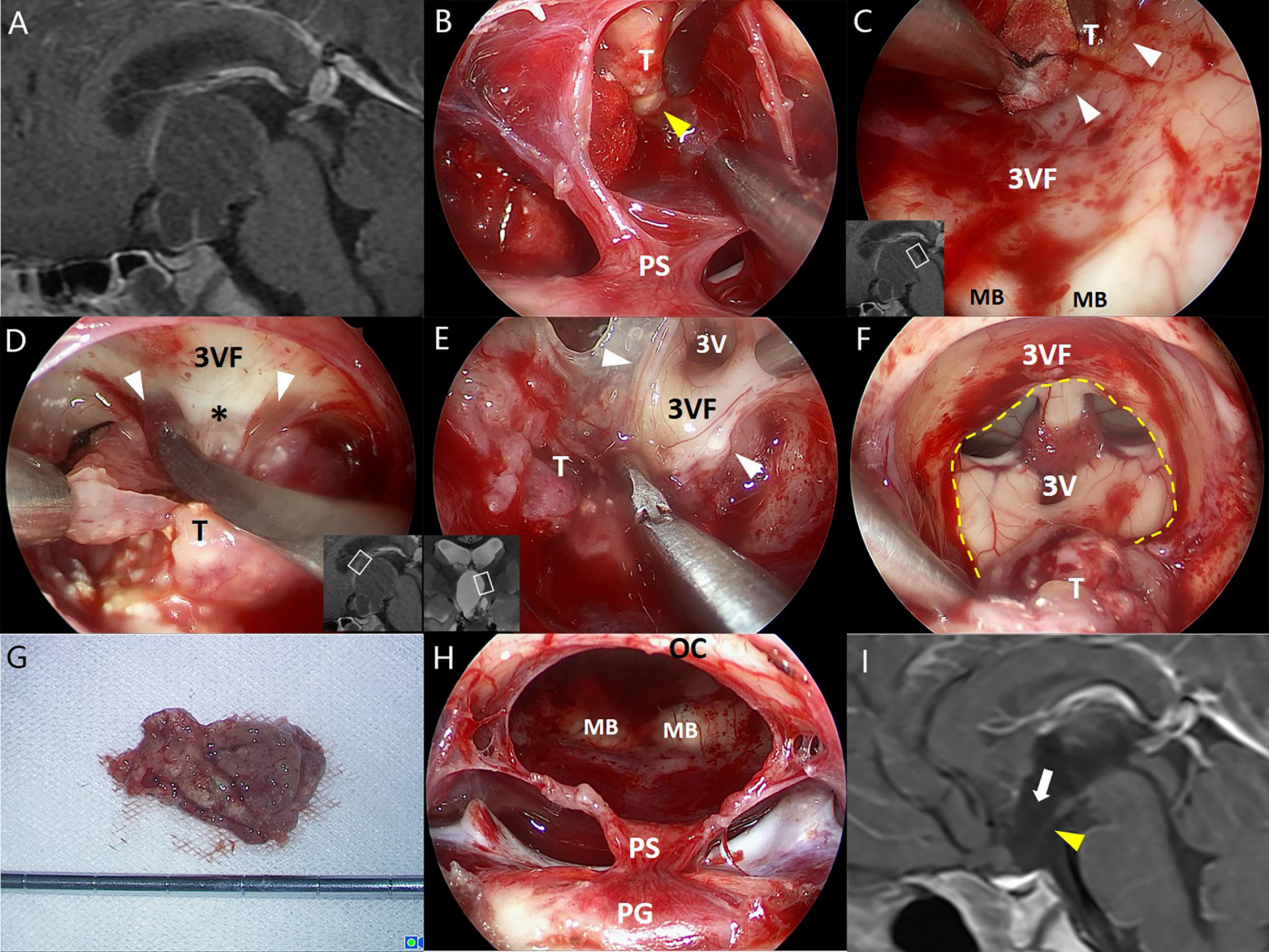
Illustrative endonasal case 2 clarifying the tumor–ventricle relationship. In **(A)**, a CP with predominantly ventricular involvement was identified on preoperative MRI. In **(B)**, the thin layer of pars tuberalis was incised longitudinally and careful dissection was performed along the tumor–infundibulum interface (yellow arrowhead) to preserve the continuity of the stretched pituitary stalk. In **(C)**, a cleavage pial plane (white arrowhead) ensured the successful dissection of the tumor and the integrity of adjacent third ventricle floor remained. In **(D)**, the pia mater was absent at some areas of the tumor–ventricle interface, while a layer of reactive gliosis (asterisk) could provide a safe cleavage plane for surgical dissection instead. In **(E)**, a clear boundary between the tumor and the third ventricle floor was observed at the lateral folder of the third ventricle, although partial remnants of the third ventricle floor was removed with the tumor due to the dense adhesion. In **(F)**, most portions of the tumor capsule were dissected with an opening left at the third ventricle floor (dashed line). In **(G)**, the tumor was removed en bloc after dissection. In **(H)**, the stretched thin layer of the pituitary stalk was mostly preserved after tumor removal. In **(I)**, postoperative MRI reveals the defect of anterior third ventricle floor (white arrow) and the stretched thin pituitary stalk (yellow arrowhead). 3V, the third ventricle; 3VF, the third ventricle floor; MB, mamillary body; OS, optic chiasm; PG, pituitary gland; PS, pituitary stalk; T, tumor.

**Figure 3:**
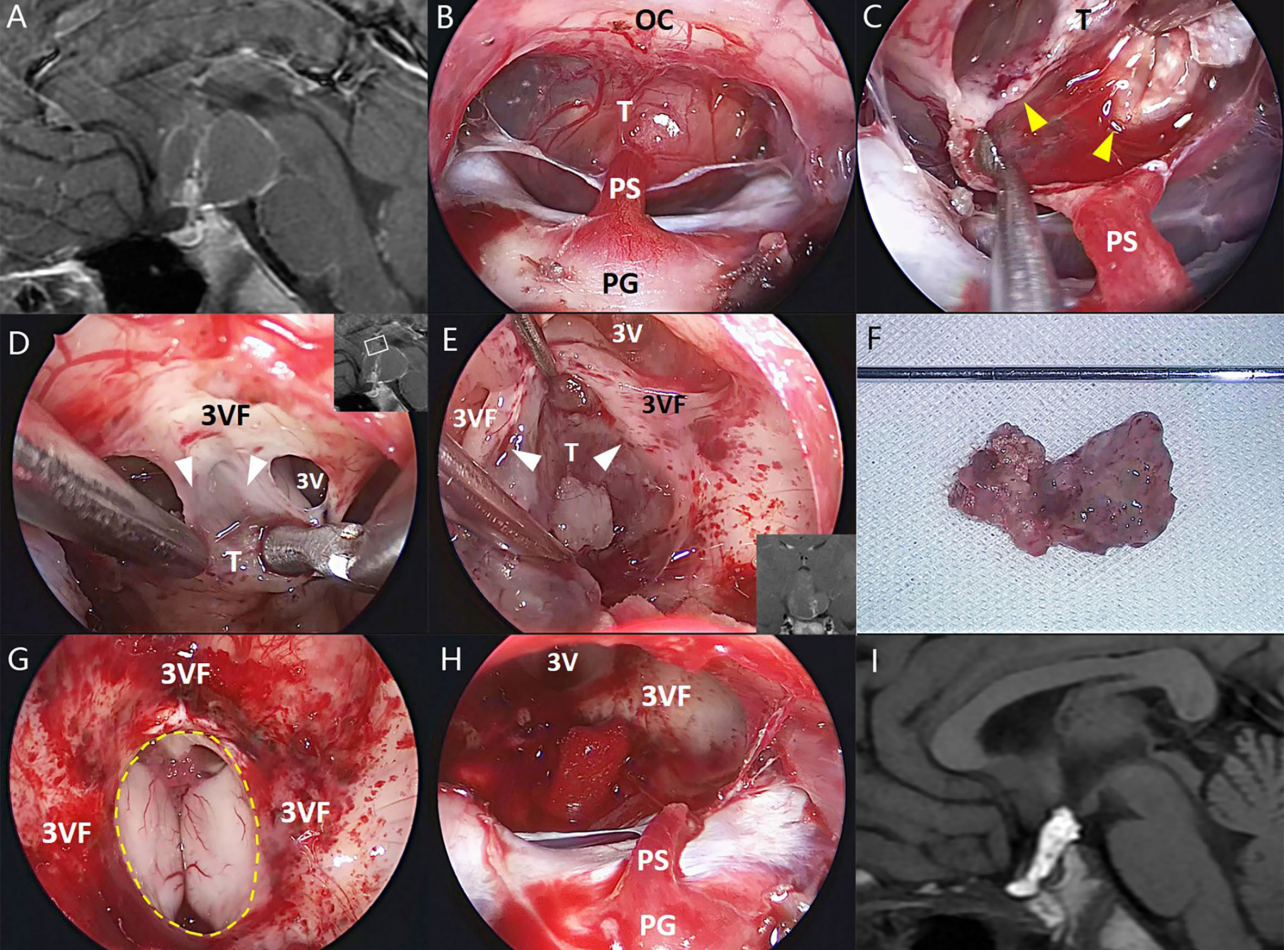
Illustrative endonasal case 3 clarifying the tumor–ventricle relationship. In **(A)**, preoperative MRI shows a tumor with predominantly ventricular development above the pituitary stalk. In **(B)**, intraoperative photograph indicates the mostly intact pituitary stalk beneath the tumor. In **(C)**, the surgical dissection was performed along a clear tumor–infundibulum interface (yellow arrowhead). In **(D, E)**, a non-functional layer of reactive gliosis between the tumor and the third ventricle floor without leptomeningeal tissue (white arrowhead) was used in this case as a safe plane of dissection, although it is difficult to remain the integrity of the third ventricle floor at some areas associated with extremely stretched thin ventricular floor or tight attachment. In **(F)**, the tumor was removed en bloc after dissection. In **(G, H)**, a moderate defect of the third ventricle floor (dashed line) and the remnants of the stretched pituitary stalk were preserved after tumor removal. In **(I)**, postoperative MRI demonstrates the total tumor removal, and the hyperintense T1 signal represents the fat used for skull base reconstruction. 3V, the third ventricle; 3VF, the third ventricle floor; OS, optic chiasm; PG, pituitary gland; PS, pituitary stalk; T, tumor.

**Figure 4:**
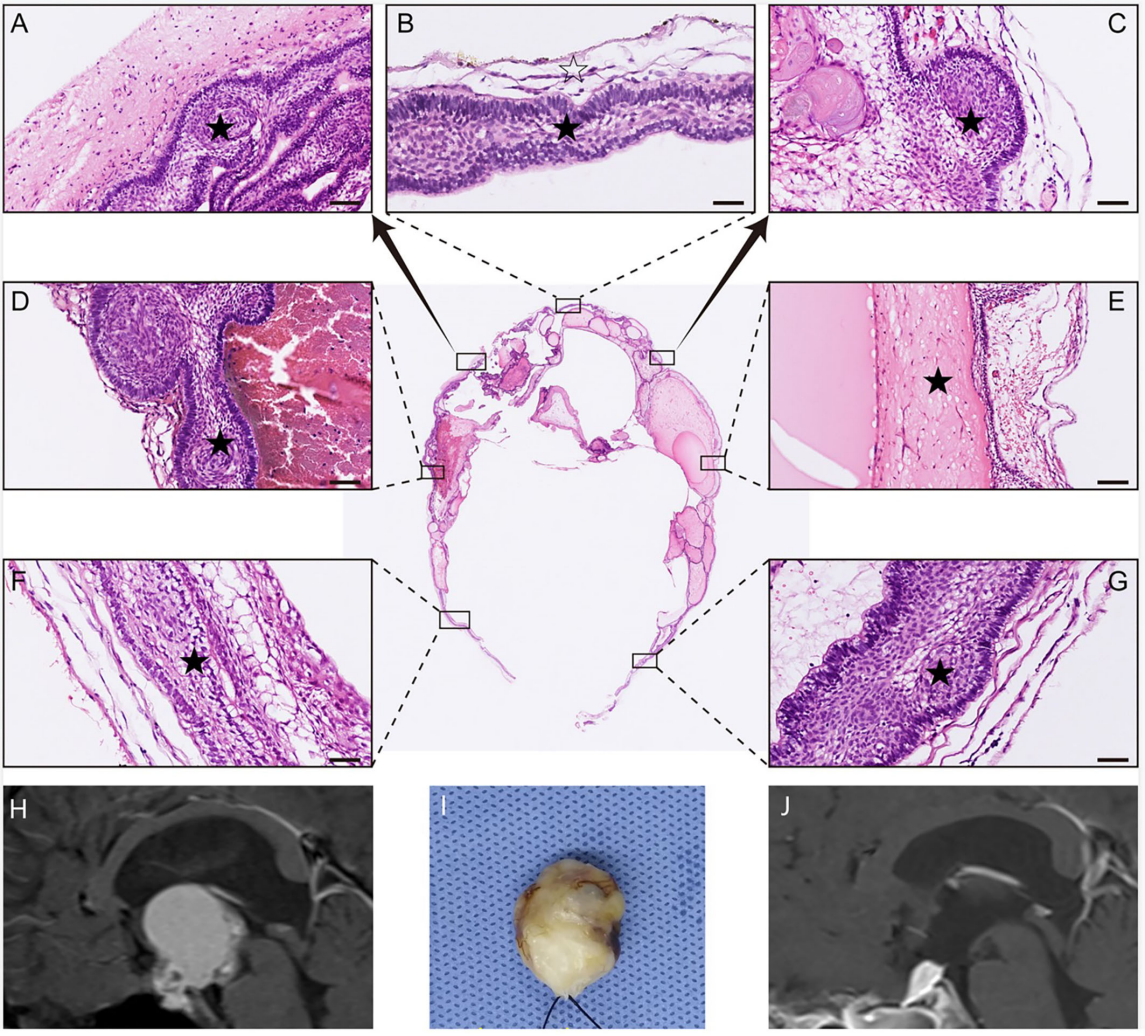
Histological examination of a remodeling tumor with mainly ventricular involvement. In **(A–G)**, multiple portions of the tumor sample were stained with HE for histopathological examination. The result of HE staining shows that the whole tumor (black pentagram) was resected along either a dense band of gliosis or some membranous structures (white pentagram). In **(H)**, preoperative MRI indicates the tumor primarily involves the third ventricle. In **(I)**, the photograph showed the gross appearance of the remodeling tumor by packing with cotton after an en bloc tumor removal. In **(J)**, postoperative MRI demonstrates the total tumoral resection, and the hyperintense T1 signal represents the fat used for skull base reconstruction.

All patients with CPs treated by endoscopic endonasal surgery at our institution between January 2017 and March 2021 were retrospectively reviewed. Patients with recurrent tumors and/or who underwent radiotherapy were excluded to avoid incorrect identification of the tumor–ventricular relationship. Preoperative imaging was reviewed in each case to evaluate the location and growth pattern of the tumor in determining the potential for study inclusion. According to our QST classification based on tumor origin as reported previously (21, 22), all tumors were classified into three types: infrasellar/subdiaphragmatic CPs (Q-CPs), subarachnoidal CPs (S-CPs), and pars tuberalis CPs (T-CPs). Confirmation of a CP with predominantly ventricular involvement was then performed by concordant assessment of three senior neurosurgeons. According to the criteria described in previous publications (2, 4, 8, 9, 14), the tumor was characterized as a CP with predominantly ventricular involvement if its main body occupy the compartment of the third ventricle on preoperative imaging, above an identifiable, almost or partially intact pituitary stalk, with or without the lower pole of the tumor slightly extending into the suprasellar area (**Figures 1A**, **2A**, **3A**, **4H**).

All data were collected from a prospectively maintained database. Each enrolled patient’s clinical chart was studied to categorize age, gender, presenting symptoms, imaging data, and surgical outcomes (**Table 1**). The tumor size was determined by the maximum diameter on preoperative sagittal or coronal MRI. Tumor consistency (solid, cystic, or mixed) was also identified on preoperative MRI. Extent of resection was determined by immediate MRI review within 48 h after surgery by an independent neuroradiologist. Accordingly, gross total resection (GTR) was defined as 100% macroscopic tumor resection, near-total resection (NTR) was defined as ≥95% but <100% resection, subtotal resection (STR) was defined as ≥80% but <95% resection, and partial resection was defined as <80% resection.

**Table 1:** Clinical chart of each enrolled patients.

Case No.	Age (years), Sex	Preoperative Presentation				Surgical Outcomes			Postoperative Outcomes		
		Symptoms	Hypothalamic Disturbance	Tumor Size^*^ (mm)	Tumor Consistency	Histological Type	EOR	Defect of 3VF	Follow-up (months)	Hypothalamic Status	Recurrence
1	41, F	HA, HP	No	23	Mixed	AD	GTR	Yes	27	Unchanged	No
2	38, F	VD	No	37	Cystic	AD	GTR	Yes	24	Worsened	No
3	41, F	HA, HP	Yes	42	Mixed	AD	GTR	Yes	17	Unchanged	No
4	16, M	HP	No	25	Mixed	AD	GTR	Yes	3	Unchanged	No
5	63, F	HA	No	15	Mixed	AD	GTR	Yes	15	Unchanged	No
6	48, M	VD	No	38	Cystic	PAP	GTR	No	54	Unchanged	No
7	33, M	HA, DI	Yes	47	Mixed	AD	GTR	Yes	9	Worsened	No
8	2, F	HA	Yes	28	Mixed	AD	GTR	Yes	36	Worsened	No
9	53, M	VD	Yes	43	Mixed	AD	GTR	Yes	47	Unchanged	No
10	50, M	HP	No	28	Mixed	AD	GTR	Yes	24	Unchanged	No
11	55, M	HA	No	19	Cystic	PAP	GTR	Yes	35	Unchanged	No
12	61, F	VD	No	51	Mixed	AD	GTR	Yes	7	Unchanged	No
13	47, F	HA, VD	Yes	57	Mixed	AD	NTR	Yes	11	Unchanged	Yes
14	52, M	HA, VD, HP	No	46	Cystic	AD	GTR	Yes	5	Worsened	No
15	57, M	HA	Yes	48	Solid	PCP	GTR	Yes	28	Improved	No
16	48, F	HA	No	35	Mixed	AD	GTR	Yes	45	Unchanged	No
17	73, M	HA	No	36	Mixed	AD	GTR	Yes	22	Worsened	No
18	45, M	VD	Yes	42	Solid	PAP	GTR	Yes	27	Improved	No
19	45, F	VD	No	45	Mixed	AD	GTR	Yes	32	Unchanged	No
20	29, F	HA	No	33	Mixed	AD	GTR	Yes	28	Worsened	No
21	55, M	HA	Yes	37	Cystic	AD	GTR	Yes	36	Improved	No
22	54, M	VD, HP	No	39	Mixed	AD	NTR	Yes	24	Worsened	No
23	47, M	HA	No	28	Mixed	AD	GTR	Yes	21	Unchanged	No
24	44, F	HA	No	35	Solid	PAP	GTR	No	33	Unchanged	No
25	49, M	VD	Yes	48	Cystic	AD	GTR	Yes	9	Worsened	No
26	58, M	VD, HP	No	39	Mixed	AD	GTR	Yes	10	Worsened	No

3VF, the third ventricle floor; AD, adamantinomatous; DI, diabetes insipidus; EOR, extent of resection; GTR, gross total resection; HA, headache; HP, hypopituitarism; NTR, near-total resection; PAP, papillary; VD, visual deterioration.

*The tumor size was determined by the maximum diameter on preoperative sagittal or coronal MRI.

### Surgical Procedure

The surgical procedure of each individual CP case was recorded by HD video and carefully reviewed after dura opening, focusing on the dissection of the tumor–brain interface. A standard endoscopic endonasal transtuberculum approach was used for tumor resection in all cases as described elsewhere. A thin translucent membrane formed by the stretched par tuberalis can be found overlying the tumor. Careful dissection and preservation of the membrane is essential to maintain the continuity of the hypothalamus–pituitary axis to maximize the possibility of recovery of postoperative hormonal function. Therefore, a longitudinal incision along the infundibulum was made in the membrane, and gentle dissection was then performed between the tumor and the membrane.

Tumors with cystic component were emptied before resection to gain better exposure and sufficient manipulating space. For large purely solid lesions, the decompressive intratumor debulking was necessary while the intact tumor capsule should be preserved. A combination of blunt and sharp extracapsular dissection was then performed to dissect the tumor away from the third ventricle floor/hypothalamus. During the dissection, the interface between the tumor and the third ventricle was carefully identified under the close-up endoscopic visualization, in an effort to maximally preserve the integrity of the third ventricular floor/hypothalamus and minimize the risk of leaving unnoticed tumor remnants behind. Finally, the surgical cavity was examined using angled endoscope after tumor removal to evaluate the integrity of the third ventricular floor/hypothalamus, and further validate tumor–third ventricle relationships.

### Histopathologic Assessment

The wholly removed tumor specimens of each case were sent for histological evaluation to identify the pathological type and investigate the tumor–third ventricle relationships. For the tumor area with dense adhesions to the third ventricular floor and unlikely to be separated from each other, the involved tissue of the third ventricle floor was removed with the tumor to ensure complete resection. As described in our previous studies, the different portions of tumor capsule in contact with the third ventricle were marked during surgery, and each marked specimen was histologically examined separately to provide a comprehensive synthesis of overall information regarding the relationships between the tumor and third ventricle.

H&E, immunochemical, and immunofluorescence staining were performed as described previously (22, 23). The antibodies used were Neurofilament (NF) (1:400, Abcam, ab7794), Glial Fibrillary Acidic Protein (GFAP) (1:200, Abcam, ab7260); pan Cytokeratin (Pan-CK) (1:300, Abcam, ab215838), Laminin β1 (1:400, Sigma, MAB1921P), CK5/6 (1:400, Sigma, SAB5600242), and Oxytocin (OXT) (1:400, Abcam, ab212193). NF was selected to mark the axon of neuron; GFAP was used to mark the gliosis; Pan-CK and CK 5/6 were used to mark the tumor; Laminin β1 was used to mark the pia mater; OXT was used to mark the secretion and transport of oxytocin in the hypothalamus.

## Results

### Patient and Tumor Characteristics

During the period from January 2017 to March 2021, a total of 223 patients with CPs underwent resection by endoscopic endonasal surgery. Of these, 78 patients with recurrent tumor and/or radiotherapy were excluded from the analysis. Accordingly, 145 patients with primary CPs were classified into three subtypes according to QST classification: 46 patients were identified with Q-CPs, 23 with S-CPs, and 76 with T-CPs. Among the 76 patients with T-CPs, 26 (34.2%) identified to have CPs with predominantly ventricular involvement were enrolled in this study. A mostly intact pituitary stalk above the tumor could be identified in 15 cases (57.7%) on preoperative sagittal or coronal MRI. The average age of the patients was 46.3 ± 14.2 years (range 2–73 years), and 11 (42.3%) of the patients were female. The maximal tumor diameter on preoperative MR images averaged 37 mm (range 15–57 mm). In regard to consistency, mixed (solid-cystic) tumors were noted in 17 cases (65.4%), purely cystic in 6 cases (23.1%), and purely solid in 3 cases (11.5%), respectively.

### Surgical Findings

GTR was achieved in 24 of 26 patients (92.3%), and NTR was noted in the remaining 2 patients. During the dissection of the tumor capsule, a circumferential, easily recognizable layer of stretched third ventricle floor was always detected surrounding the tumor. In most areas of the tumor–brain interface, the tumor capsule was usually easy to strip away from the third ventricle floor/hypothalamus upon a clear plane of the pia mater or gliosis. By contrast, in some areas (mostly at the central area) of the tumor–brain interface, it is difficult to remove the tumor capsule without trespassing the ventricle floor due to extremely thin floor and/or dense adhesion. Therefore, the tightly adhered portions of the tumor were removed together with adjacent third ventricle floor, leaving an opening with various size at the floor in most cases (**Figures 1**–**3**). Only two patients (7.7%) with moderate tumor size had the intact third ventricle floor after tumor removal. The infundibulum was mostly preserved with pituitary–hypothalamus continuity in 12 patients (46.1%), partially preserved in 8 patients (30.8%), and sacrificed in 6 patients (23.1%) due to extremely thin fiber remnants.

### Histopathological Results

**Figure 5:**
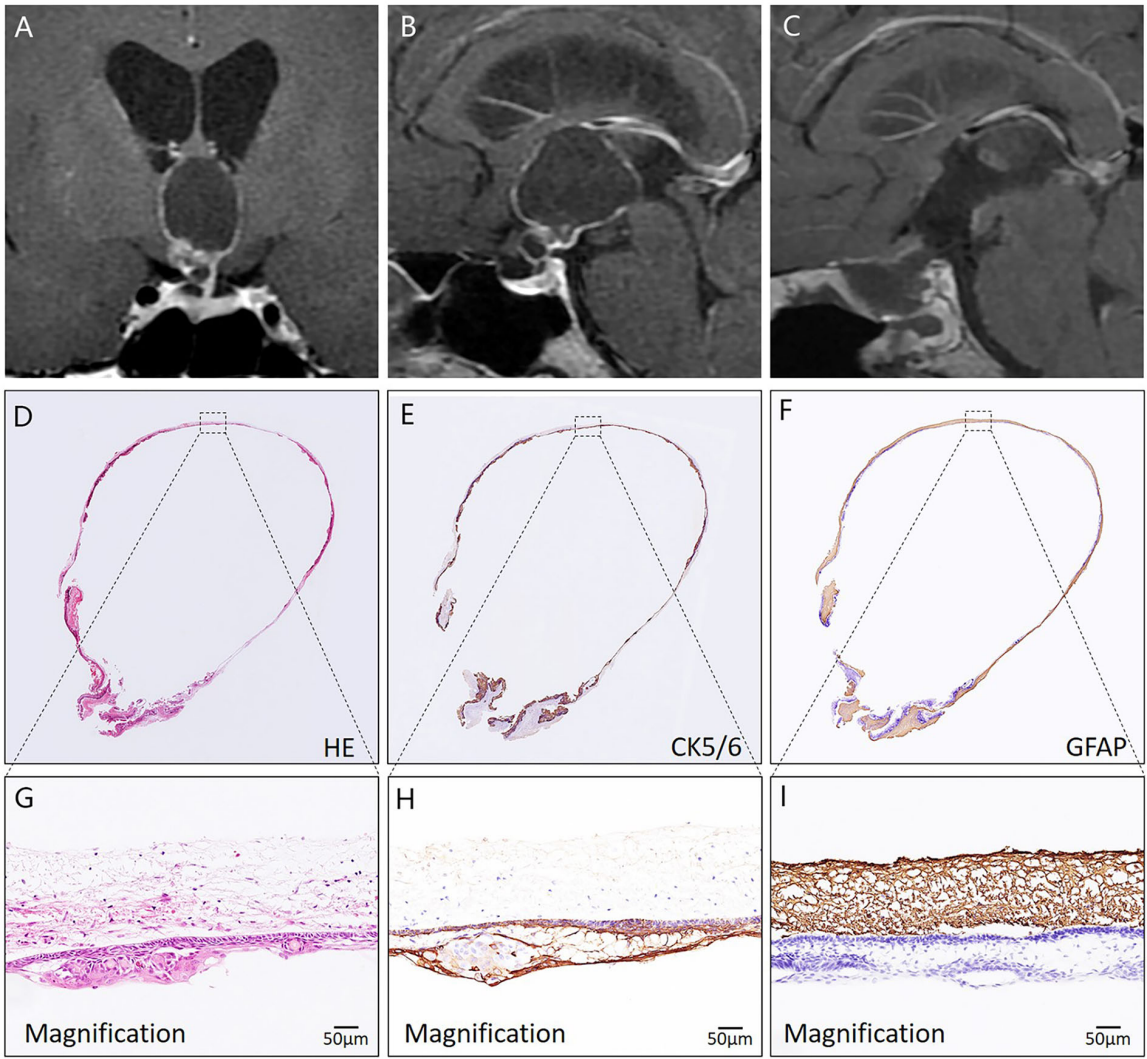
Histological assessment of a CP with predominantly ventricular involvement using HE and immunohistochemical staining. In **(A, B)**, preoperative coronal and sagittal MRI reveals that a cystic-solid mass mainly develops into the compartment of the third ventricle. In **(C)**, postoperative MRI demonstrates complete tumor removal. In **(D–I)**, the pathological image of the tumor specimen indicates that the whole tumor (CK5/6 positive) was resected along a dense band of gliosis (GFAP positive).

**Figure 6:**
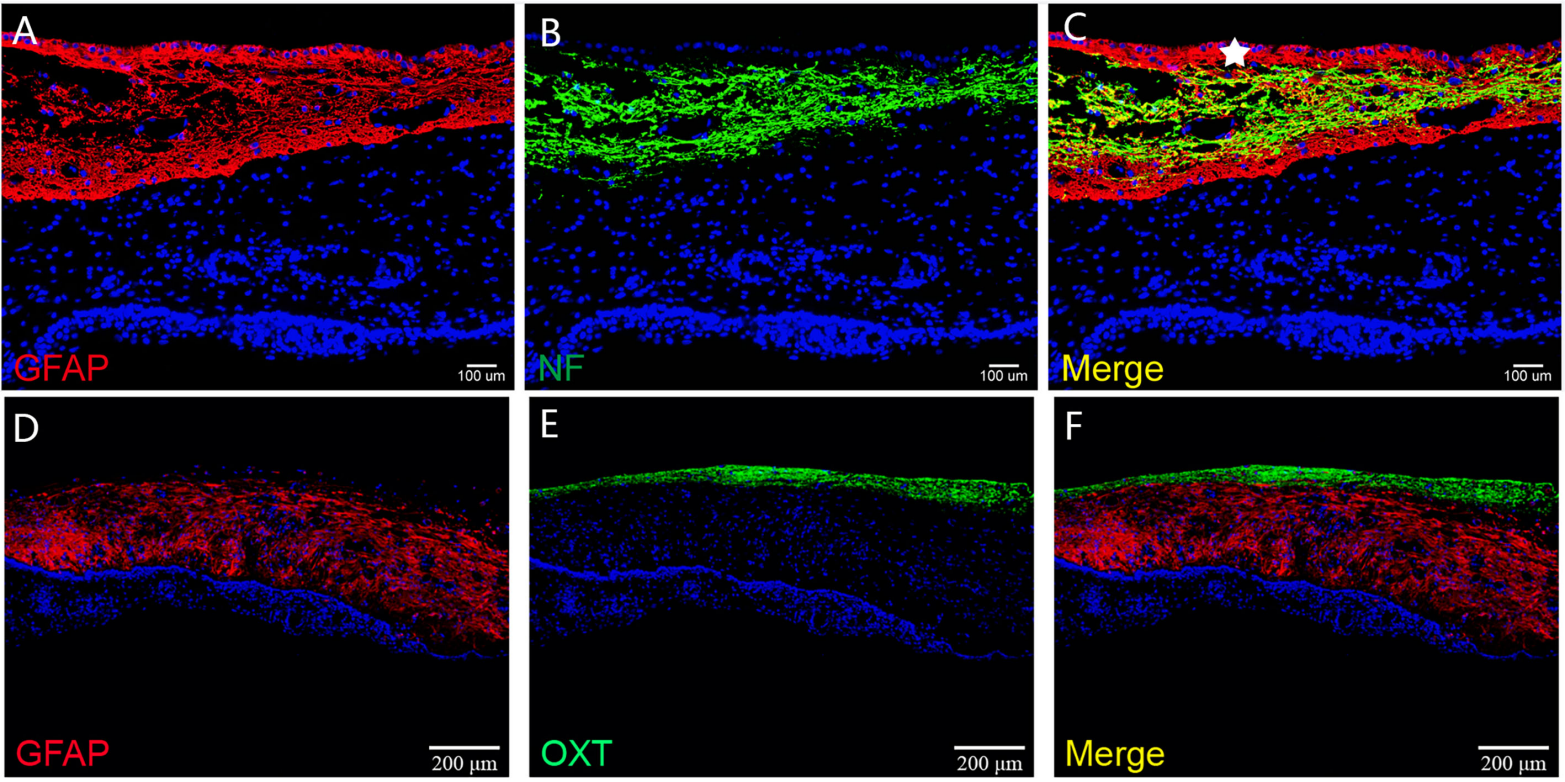
The histological architecture between the tumor and the third ventricular floor. In **(A–F)**, immunofluorescent staining shows a layer of gliosis (GFAP positive) with various thickness between the tumor and the neural tissue of the third ventricle floor. The neural tissue of the third ventricle floor/hypothalamus (NF and OXT positive) can be observed on the outer border of the gliosis. The ependymal cells of the third ventricle (white pentagram) could be found in some samples, which indicated the extraventricular topography of CP.

According to pathological reports, adamantinomatous CPs were the predominant type, noting in 21 cases (80.8%). H&E and immunofluorescence staining on marked portions of tumor capsule involving third ventricle showed that the pia matter was frequently detected at most of the tumor–brain interface, except at the antero-basal border of tumor contacting with the third ventricle floor, which is often near the presumed site of tumor origin. In this situation, a layer of gliosis with various thickness was instead observed between the tumor and the neural tissue of the third ventricle floor (**Figures 4**–**6**).

**Figure 7:**
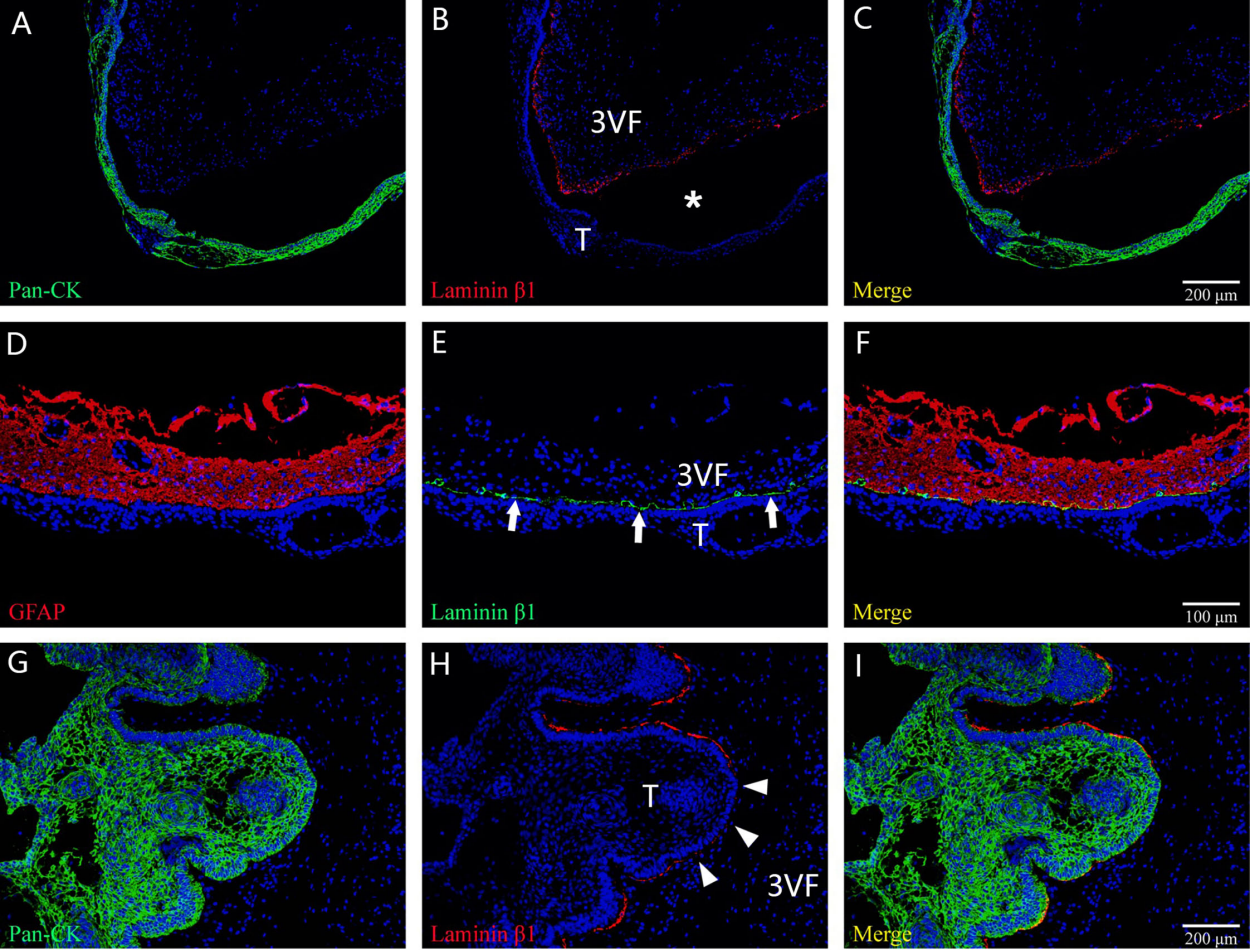
The main histological patterns found for the tumor–ventricle floor interface among tumors with a predominant ventricular involvement. In **(A–C)**, immunofluorescent staining shows a moat-like structure in which a gap (white asterisk) is detected between the tumor and third ventricle floor with the presence of the intact pia mater. In **(D–F)**, immunofluorescent staining indicates a beach-like structure (white arrow) characterized by a smooth boundary at the tumor–ventricle interface without interposed gap, and the pia mater of the third ventricle floor is intact. In **(G–I)**, immunofluorescent staining demonstrates a finger-like structure in which the tumor forms a finger-like bulge in the third ventricle floor, with the absence of the pia mater at some areas (white arrowhead) and the development of gliosis between the tumor and the neural tissue of the third ventricle floor. 3VF, third ventricle floor; T, tumor.

Collectively, three morphological patterns between the tumor and the third ventricle floor were noted in this series: (1) the moat-like pattern, in which the pia matter was intact at the tumor–brain interface and a gap was found between the tumor and the pia mater; (2) the beach-like pattern, in which there existed a continuous smooth plane between the tumor and the ventricle floor without any gap, and the intact pia mater was still detected at the tumor–ventricle interface; and (3) the finger-like pattern, in which the tumor formed finger-like structures that invaded the third ventricle floor, with the absence of the pia mater and the development of reactive gliosis between the tumor and the neural tissue of the third ventricle floor (**Figure 7**).

## Discussion

### Study Inclusion

CPs predominantly involving the third ventricle are not common while posing the greatest surgical challenge of all CPs due to their intimate anatomical and functional relationships with the hypothalamus. In the late 1920s, Cushing first realized that some CPs had an “intraventricular” position (24). In 1985, Steno (25) demonstrated an “intraventricular” subset among suprasellar CPs by stereoscopic and microscopic investigation in 30 autopsies. Since then, the term “intraventricular” was widely used by many authors as a subset of CPs in their classification schemes. Yasargil et al. (13) classified CPs into five subtypes in their surgical series of 144 cases and type f was assigned to define “purely intraventricular” CPs, which was described as “lying within the third ventricle”. Kassam et al. (7) described tumors isolated to the third ventricle as “intraventricular” (type IV) CPs in their surgical series using an endoscopic endonasal approach. Pascual et al. (26) analyzed 130 CPs previously described as “intraventricular” in various publications, and further recategorized four subtypes: (1) “strictly intraventricular”, (2) “not strictly intraventricular”, (3) “secondarily intraventricular”, and (4) “pseudointraventricular”. In categorizing the tumors in this paper, tumors predominantly occupying the compartment of the third ventricle that satisfied the Pascual criteria of “strictly intraventricular” and “not strictly intraventricular” were selected for study inclusion, which is consistent with Forbes et al.’s study (6). According to our QST classification, the enrolled 26 cases belonged to the subset of T-CPs, which were presumed to arise in the top of the par tuberalis. The specific originating site should be primarily responsible for the predominantly upper extension of such tumors, and the purpose of this study is to reveal the real relationship between the tumor and the third ventricle floor.

### Endoscopic Endonasal Surgery for Intraoperative Investigation

In the era of micro-neurosurgery, the basic knowledge regarding the tumor–brain relationship of CPs was mainly established based on microscopic findings. The investigation of the delicate anatomy of the hypothalamic–pituitary axis was limited by poor visualization. During the last two decades, the refinement of the endoscopic endonasal surgery has significantly changed the management of CPs. This approach can provide direct visualization of the retrochiasmatic space and is especially suitable for the exposure of the third ventricle undersurface (17, 19, 20). Additionally, close-up observation provided by endoscope and great advance of visualization technologies allows for gaining more anatomical and detailed information of this intricate area. The CP classification proposed by Kassam et al. (7) in 2008 was established based on the relevant relationship between the tumor and the pituitary stalk observed through the endoscopic transsphenoidal approach, although the visual resolution of endoscopy seemed not satisfactory according to current standard. Despite the controversy regarding the approach selection of “intraventricular” CPs between the transcranial and the endonasal approach, it is undoubted that the latter may provide optimal visualization of the third ventricle floor. Therefore, we intentionally reviewed the surgical procedure of such CPs treated by endoscopic endonasal approach in this series, in an attempt to present more anatomical evidence illuminating the tumor–ventricle relationships compared with traditional microsurgery.

### Surgical Findings at the Tumor–Ventricle Interface

Although the tumors appeared to be “intraventricular” on preoperative MRI, a circumferential, well-defined layer of stretched third ventricle floor was always observed surrounding the tumors involving the third ventricle during the surgical dissection. Most portions of the tumor capsule could be easily stripped away from the third ventricle floor along a cleavage plane of the pia mater or the gliosis, except at some points with tight adherence or extremely thin remnants of the stretched third ventricle floor. These points were probably associated with the origin of the tumor. The findings strongly suggest that the progressive upward growth of the tumor originally developing outside the floor of the third ventricle would cause a circumferential layer of the stretched floor surrounding the tumor and finally occupy the compartment of the third ventricle. Therefore, such tumors should be regarded as having an extraventricular origin instead of a primary intraventricular development. The proposal can be strengthened by the fact that two patients in this series had the intact third ventricle floor after tumor removal (**Figure 1**).

We found that a wide defect of the third ventricle floor after tumor resection occurred more commonly in those cases with large tumor, and an intact or small deficient third ventricle floor was usually observed in the cases with papillary CPs, suggesting that the extent of ventricular floor preservation may be related to tumor size and pathological type. Adamantinomatous CPs seemed more likely to cause tight adhesion between the tumor and the third ventricle floor/hypothalamus. Some authors observed that the defect left in the floor of the third ventricle after tumor removal did not necessarily correlate to iatrogenic hypothalamic injury (27). The possible explanation is that the functional hypothalamic nucleus was displaced bilaterally with the progressive growth of the tumor, leaving a nonfunctional tissue layer of the third ventricle floor overlying the dome of the tumor.

### Histological Relationships Between the Tumor and the Third Ventricle

Among the ill-defined category of “intraventricular” CPs, a group of infundibulo-tuberal lesions were previously characterized as those replacing the third ventricular floor, above an identifiable, almost intact pituitary stalk, and whole mass is occupying partial or the entire third ventricle chamber (11, 18). CPs with such a topography were thought to originate within the neural tissue of the third ventricle floor and then progressively grow into the ventricle cavity (15, 16). The above conclusive demonstration on the topographical relationships of tumors with the third ventricle were established on basis of numerous previously reported necropsy or pathohistological studies (11). In these studies, most of intraoperative tumor specimens were obtained *via* piecemeal resection, and more importantly, the accurate investigation regarding the components of multiple layers of tissue in contact with tumors was absent. Therefore, previous evidence was inadequate to support the address of “intraventricular” or “subpial” topography of such CPs. In this series, we used H&E and immunochemical/immunofluorescent staining to precisely identify different layers of tissue in contact with tumors in histological samples taken from wholly removed lesions. We believed that it may provide a more explicit and powerful evidence for investigating the real tumor–ventricle relationships of CPs with predominantly ventricular involvement.

Our histological results revealed that the pia mater was detected overlying most of the tumor–brain interface in the tumors predominantly involving the third ventricle, regardless of tumor size or tomography. The pia mater may be absent at some areas of the interface, while a layer of reactive gliosis with various thickness was instead observed between the tumor and the neural tissue of the third ventricle floor, which is consistent with previous descriptions (18, 28–30). Moreover, a thin layer of neural tissue and ependyma was also identified on the outer border of the gliosis. Early in 1904, Erdheim also observed a thin layer of stretched neural tissue corresponding to the remnant of the third ventricle floor enveloping circumferentially the tumor in his cadaveric study of a patient with CPs apparently strictly confined within the third ventricle (31, 32). Our findings further confirmed that several layers of tissue constituting the floor of third ventricle covers the dorsal aspect of the CPs with ventricular involvement, and such tumors should not be defined as “intraventricular” lesions.

According to the literature (3, 31, 33), CPs involving third ventricle are presumably originated from nests of epithelial cells incorporated or derived from metaplastic transformation of glandular cells of the pars tuberalis of the pituitary gland. The reason for predominantly ventricular development of these tumors remains unknown. Ciric and Cozzens (34) attributed it to the tumor origin within the neural tissue of the third ventricle floor before the formation of the leptomeningeal layer of arachnoid–pia mater. However, the theory cannot explain the fact observed in our histological investigation that the main body of the tumors was located outside the pia mater of the third ventricle floor. Interestingly, in our previous surgical cases of CP involving the third ventricle, bundles of dense arachnoid trabeculae were frequently observed surrounding the infundibulo-tuberal area, which tends to form a firm barrier preventing the tumor from extending ventrally. Aguado et al. (35) also described in their study that the pars tuberalis is perforated by subarachnoid channels. Therefore, we postulate that the predominantly intraventricular development of such tumor may be associated with its originating site at the uppermost area of the pars tuberalis combining dense trabecular barrier at the point of infundibulo-tuberal area. Nevertheless, further evidence regarding the relationship between the characteristics of arachnoid trabeculae and the tumor genesis is required to validate the hypothesis.

Three morphological relationships of the tumor to the third ventricular floor based on histological assessment were summarized in this series: moat-like, beach-like, or finger-like. The moat-like and beach-like patterns had intact pia mater and could provide a clear cleavage plane of dissection, in which the integrity of the third ventricle floor/hypothalamus was expected to be achieved after tumor resection. For the finger-like pattern, a band of reactive gliosis with various thickness instead of the pia mater separated tumor from the neural layer of the third ventricle floor. The tight tumoral adherence at some areas was thought to be related to the lack of a leptomeningeal layer separating the neural tissue from the tumor wall, and a reactive gliosis layer will develop between the lesion and the hypothalamic nuclei during the progressive tumor development (10, 30).

The functionality of the layer overlying the dome of CPs with predominantly ventricular involvement remains controversial. Some authors warned about the risk of trespassing this still viable layer through a transventricular or a translaminar terminalis approach, while others affirmed the nonfunctional gliotic nature of such tissue, which provides a safe cleavage plane for surgical dissection of the lesion (11, 27). According to our histological assessment, the pia mater or gliosis layer separated the tumor from neural tissue of the third ventricle floor and may provide a safe dissection plane for most tumors. However, at some areas with thin gliosis layer, it is difficult to remove the tumor without trespassing the adjacent neural tissue due to the extreme thinness of the third ventricle floor and the dense adhesion in between.

Multiple factors, including the heterogeneity of the reactive gliosis, the thickness of the stretched ventricular floor, the adhesiveness of tumor interface, and possible displaced hypothalamic nuclei, were associated with the difficulty of tumor dissection and high risk of damaging the adjacent functional neural tissue. Landolt (36) found in his electron microscopic study on the boundaries of CPs that the basal membranes of epithelial and reactive glia were separated by a few collagen fibers, providing a safe cleavage plan of dissection for neurosurgeon. However, the gliosis boundary had a heterogeneous, variable thickness even in the same tumor, and the existence of a gliosis layer separating the tumor from hypothalamus could not guarantee the safety of surgical dissection. Taken together, three possibilities may occur during surgical dissection, the presence of an easily dissectible plane separated by the pia mater, an identifiable functionless interface composed of thick reactive gliosis, or the lack of a safe manipulable cleavage plane associated with the extremely thin gliosis layer and dense adhesion.

Prevention of hypothalamic injury remains as the neurosurgeon’s principal concern during removal of CPs with predominantly ventricular involvement. Awareness of not real “intraventricular” nature histologically for these lesions would predict that the cleavage plane of dissection will be circumferential surrounding the tumor, and remind neurosurgeons of carefully identifying the plane and performing delicate dissection along the safe surgical plane of the tumor–ventricle interface, instead of the “blind” pulling of the tightly adhered portions of the tumor. In other words, the surgical and histological findings in this study regarding the tumor–third ventricle floor relationship in CPs with predominantly ventricular involvement could provide theoretical evidence for the attempt of total tumor resection while minimizing the risk of hypothalamic injury.

## Conclusion

CPs with predominantly ventricular involvement were separated from the third ventricle floor by the intact pia mater at most of the tumor–brain interface. Histologically, these tumors should not be considered as “intraventricular” or “subpial” lesions. The pia mater could be absent at some areas where a layer of reactive gliosis with various thickness developed instead between the tumor and the neural tissue of the third ventricle floor. On the basis of surgical and histological results, we postulated that these tumors originate from the top of par tuberalis, then develop a progressive growing dorsally, which would cause a circumferential layer of the stretched third ventricle floor surrounding the tumor, and finally occupy the third ventricle chamber. Awareness of not real “intraventricular” nature histologically for these lesions would predict the circumferential cleavage plane of dissection surrounding the tumor, and remind neurosurgeons of performing delicate dissection along the safe surgical plane of the tumor–ventricle interface to achieve total tumoral resection with minimized risk of hypothalamic damage.

## Data Availability Statement

The original contributions presented in the study are included in the article/supplementary material. Further inquiries can be directed to the corresponding author.

## Author Contributions

Conception and design: JF and YL. Acquisition of data: YL, YB, ZF, JN, BQ, and JPe. Analysis and interpretation of data: YL and CW. Histopathological assessment: YL and CW. Drafting the article: JF. Critically revising the article: JF. Reviewed submitted version of manuscript: SQ. Approved the final version of the manuscript on behalf of all authors: SQ. Administrative/technical/material support: JPa and YP. Study supervision: SQ. All authors contributed to the article and approved the submitted version.

## Funding

This study was supported by grants from the Science and Technology Program of Guangdong (2016A020213006, 2017A020215048, and 2017A020215191), the Natural Science Foundation of Guangdong (2016A030310377), the Science and Technology Program of Guangzhou (201707010149), and the President Foundation of Nanfang Hospital, Southern Medical University (2015C018, 2016L002, 2016B006, and 2017Z009). The authors have no other personal financial or institutional interest in any of the drugs, materials, or devices described in this article.

## Conflict of Interest

The authors declare that the research was conducted in the absence of any commercial or financial relationships that could be construed as a potential conflict of interest.

## Publisher’s Note

All claims expressed in this article are solely those of the authors and do not necessarily represent those of their affiliated organizations, or those of the publisher, the editors and the reviewers. Any product that may be evaluated in this article, or claim that may be made by its manufacturer, is not guaranteed or endorsed by the publisher.

## References

[1] Karavitaki N Cudlip S Adams CB Wass JA. Craniopharyngiomas. Endocr Rev (2006) 27(4):371–97. doi: 10.1210/er.2006-0002 16543382

[2] Wang KC Kim SK Choe G Chi JG Cho BK. Growth Patterns of Craniopharyngioma in Children: Role of the Diaphragm Sellae and Its Surgical Implication. Surg Neurol (2002) 57(1):25–33. doi: 10.1016/s0090-3019(01)00657-7 11834269

[3] Zada G Lin N Ojerholm E Ramkissoon S Laws ER. Craniopharyngioma and Other Cystic Epithelial Lesions of the Sellar Region: A Review of Clinical, Imaging, and Histopathological Relationships. Neurosurg Focus (2010) 28(4):E4. doi: 10.3171/2010.2.Focus09318 20367361

[4] Almeida JP Workewych A Takami H Velasquez C Oswari S Asha M. Surgical Anatomy Applied to the Resection of Craniopharyngiomas: Anatomic Compartments and Surgical Classifications. World Neurosurg (2020) 142:611–25. doi: 10.1016/j.wneu.2020.05.171 32987617

[5] de Lara D Ditzel Filho LF Muto J Otto BA Carrau RL Prevedello DM. Surgical Management of Craniopharyngioma With Third Ventricle Involvement. Neurosurg Focus (2013) 34(1 Suppl):Video 5. doi: 10.3171/2013.V1.Focus12330 23282158

[6] Forbes JA Ordóñez-Rubiano EG Tomasiewicz HC Banu MA Younus I Dobri GA. Endonasal Endoscopic Transsphenoidal Resection of Intrinsic Third Ventricular Craniopharyngioma: Surgical Results. J Neurosurg (2018) 131:1152–62. doi: 10.3171/2018.5.Jns18198 30497140

[7] Kassam AB Gardner PA Snyderman CH Carrau RL Mintz AH Prevedello DM. Expanded Endonasal Approach, a Fully Endoscopic Transnasal Approach for the Resection of Midline Suprasellar Craniopharyngiomas: A New Classification Based on the Infundibulum. J Neurosurg (2008) 108(4):715–28. doi: 10.3171/jns/2008/108/4/0715 18377251

[8] Lubuulwa J Lei T. Pathological and Topographical Classification of Craniopharyngiomas: A Literature Review. J Neurol Surg Rep (2016) 77(3):e121–7. doi: 10.1055/s-0036-1588060 PMC4993606 27556005

[9] Morisako H Goto T Goto H Bohoun CA Tamrakar S Ohata K. Aggressive Surgery Based on an Anatomical Subclassification of Craniopharyngiomas. Neurosurg Focus (2016) 41(6):E10. doi: 10.3171/2016.9.Focus16211 27903115

[10] Pascual JM González-Llanos F Barrios L Roda JM. Intraventricular Craniopharyngiomas: Topographical Classification and Surgical Approach Selection Based on an Extensive Overview. Acta Neurochir (2004) 146(8):785–802. doi: 10.1007/s00701-004-0295-3 15254801

[11] Pascual JM Prieto R Carrasco R. Infundibulo-Tuberal or Not Strictly Intraventricular Craniopharyngioma: Evidence for a Major Topographical Category. Acta Neurochir (2011) 153(12):2403–25; discussion 26. doi: 10.1007/s00701-011-1149-4 21918833

[12] Prieto R Pascual JM Hofecker V Winter E Castro-Dufourny I Carrasco R. Craniopharyngioma Adherence: A Reappraisal of the Evidence. Neurosurg Rev (2020) 43(2):453–72. doi: 10.1007/s10143-018-1010-9 30043262

[13] Yaşargil MG Curcic M Kis M Siegenthaler G Teddy PJ Roth P. Total Removal of Craniopharyngiomas. Approaches and Long-Term Results in 144 Patients. J Neurosurg (1990) 73(1):3–11. doi: 10.3171/jns.1990.73.1.0003 2352020

[14] Yu T Sun X Ren X Cui X Wang J Lin S. Intraventricular Craniopharyngiomas: Surgical Management and Outcome Analyses in 24 Cases. World Neurosurg (2014) 82(6):1209–15. doi: 10.1016/j.wneu.2014.06.015 24937597

[15] Pascual JM Carrasco R Prieto R Gonzalez-Llanos F Alvarez F Roda JM. Craniopharyngioma Classification. J Neurosurg (2008) 109(6):1180–2; author reply 2-3. doi: 10.3171/jns.2008.109.12.1180 19035739

[16] Pascual JM Prieto R Navas M Carrasco R. Conquest of Third Ventricle Craniopharyngiomas. J Neurosurg (2010) 112(5):1156–61; author reply 61. doi: 10.3171/2009.8.Jns091094 20345221

[17] de Divitiis E Cappabianca P Cavallo LM Esposito F de Divitiis O Messina A. Extended Endoscopic Transsphenoidal Approach for Extrasellar Craniopharyngiomas. Neurosurgery (2007) 61(5 Suppl 2):219–27. doi: 10.1227/01.neu.0000303220.55393.73 18091236

[18] Kubota T Yamamoto S Kohno H Ito H Hayashi M. Operative Procedures of Craniopharyngioma Estimated by Autopsy Findings (Author’s Transl). Neurol Med Chir (1980) 20(4):341–54. doi: 10.2176/nmc.20.341 6155622

[19] Gardner PA Prevedello DM Kassam AB Snyderman CH Carrau RL Mintz AH. The Evolution of the Endonasal Approach for Craniopharyngiomas. J Neurosurg (2008) 108(5):1043–7. doi: 10.3171/jns/2008/108/5/1043 18447729

[20] Laufer I Anand VK Schwartz TH. Endoscopic, Endonasal Extended Transsphenoidal, Transplanum Transtuberculum Approach for Resection of Suprasellar Lesions. J Neurosurg (2007) 106(3):400–6. doi: 10.3171/jns.2007.106.3.400 17367062

[21] Fan J Liu Y Pan J Peng Y Peng J Bao Y. Endoscopic Endonasal *Versus* Transcranial Surgery for Primary Resection of Craniopharyngiomas Based on a New QST Classification System: A Comparative Series of 315 Patients. J Neurosurg (2021) 135:1298–309. doi: 10.3171/2020.7.Jns20257 33668037

[22] Liu Y Qi ST Wang CH Pan J Fan J Peng JX. Pathological Relationship Between Adamantinomatous Craniopharyngioma and Adjacent Structures Based on QST Classification. J Neuropathol Exp Neurol (2018) 77(11):1017–23. doi: 10.1093/jnen/nly083 30239800

[23] Qi S Liu Y Wang C Fan J Pan J Zhang X. Membrane Structures Between Craniopharyngioma and the Third Ventricle Floor Based on the QST Classification and Its Significance: A Pathological Study. J Neuropathol Exp Neurol (2020) 79(9):966–74. doi: 10.1093/jnen/nlaa087 32791520

[24] Prieto R Pascual JM Barrios L. Harvey Cushing’s Craniopharyngioma Treatment: Part 2. Surgical Strategies and Results of His Pioneering Series. J Neurosurg (2018) 131(3):964–78. doi: 10.3171/2018.5.Jns18154 30497192

[25] Steno J. Microsurgical Topography of Craniopharyngiomas. Acta Neurochir Supplementum (1985) 35:94–100. doi: 10.1007/978-3-7091-8813-2_16 3867266

[26] Pascual JM Prieto R Carrasco R Barrios L. Displacement of Mammillary Bodies by Craniopharyngiomas Involving the Third Ventricle: Surgical-MRI Correlation and Use in Topographical Diagnosis. J Neurosurg (2013) 119(2):381–405. doi: 10.3171/2013.1.Jns111722 23540270

[27] Sweet WH. Radical Surgical Treatment of Craniopharyngioma. Clin Neurosurg (1976) 23:52–79. doi: 10.1093/neurosurgery/23.cn_suppl_1.52 975700

[28] Kobayashi T Kageyama N Yoshida J Shibuya N Yonezawa T. Pathological and Clinical Basis of the Indications for Treatment of Craniopharyngiomas. Neurol Med Chir (1981) 21(1):39–47. doi: 10.2176/nmc.21.39 6168949

[29] Northfield DW. Rathke-Pouch Tumours. Brain: J Neurol (1957) 80(3):293–312. doi: 10.1093/brain/80.3.293 13471802

[30] Qi S Lu Y Pan J Zhang X Long H Fan J. Anatomic Relations of the Arachnoidea Around the Pituitary Stalk: Relevance for Surgical Removal of Craniopharyngiomas. Acta Neurochir (2011) 153(4):785–96. doi: 10.1007/s00701-010-0940-y PMC3059825 21271263

[31] Erdheim J. Über Hypophysengangsgeschwulste Und Hirmcholesteatome. Sitzungsb Kais Akad Wissen Math Naturw Klin (1904) 113:537–726.

[32] Pascual JM Prieto R Rosdolsky M Strauss S Castro-Dufourny I Hofecker V. Cystic Tumors of the Pituitary Infundibulum: Seminal Autopsy Specimens (1899 to 1904) That Allowed Clinical-Pathological Craniopharyngioma Characterization. Pituitary (2018) 21(4):393–405. doi: 10.1007/s11102-018-0889-z 29680871

[33] Attwell W. The Development of the Hypophysis Cerebri in Man, With Special Reference to the Pars Tuberalis. Am J Anat (1926) 37:159–93. doi: 10.1002/aja.1000370107

[34] Ciric IS Cozzens JW. Craniopharyngiomas: Transsphenoidal Method of Approach–for the Virtuoso Only? Clin Neurosurg (1980) 27:169–87. doi: 10.1093/neurosurgery/27.CN_suppl_1.169 7273552

[35] Aguado LI Schoebitz K Rodríguez EM. Intercellular Channels in the Pars Tuberalis of the Rat Hypophysis and Their Relationship to the Subarachnoid Space. Cell Tissue Res (1981) 218(2):345–54. doi: 10.1007/bf00210349 7261033

[36] Landolt AM. The Ultrastructure of Craniopharyngioma. Schweiz Archiv Neurol Neurochir Psychiatr Arch Suisse Neurol Neurochir Psychiatr (1972) 111(2):313–29. 4653255

